# Editorial: Breaking the mental health stigma for people with substance use disorders

**DOI:** 10.3389/fpsyt.2024.1481215

**Published:** 2024-09-25

**Authors:** Richard Appiah, Ottar Ness, Kofi E. Boakye

**Affiliations:** ^1^ Department of Psychology, Faculty of Health and Life Sciences, Northumbria University, Newcastle upon Tyne, United Kingdom; ^2^ College of Health Sciences, University of Ghana, Accra, Ghana; ^3^ Department of Psychology, University of Johannesburg, Johannesburg, South Africa; ^4^ Department of Education and Lifelong Learning, Norwegian University of Science and Technology, Trondheim, Norway; ^5^ School of Criminology, University of Leicester, Leicester, United Kingdom; ^6^ Institute of Criminology, University of Cambridge, Cambridge, United Kingdom

**Keywords:** substance use disorder, stigma, mental health stigma, substance use disorder treatment and prevention, substance use disorder and mental health

Substance use disorders (SUDs) affect millions globally, posing a significant public health challenge exacerbated by pervasive stigma (1, 2). This stigma, deeply entrenched in societal attitudes, manifests at multiple levels—self, social, and structural—resulting in discrimination, reduced access to treatment, and poorer psychological outcomes (3). Empirical research explicates the harmful impacts of stigma, including diminished hope, lower self-esteem, increased psychiatric symptoms, and social isolation (4, 5). This Research Topic (RT), titled ‘*Breaking the Mental Health Stigma for People with Substance Use Disorders*’, aims to present empirical and theoretical insights that highlight the detrimental effects of stigma and propose strategies to mitigate its impact.

The ongoing work of scholars and practitioners in identifying the origins and effects of stigma related to SUDs is critical for advancing the field and improving outcomes for affected individuals. The articles in this RT, selected through rigorous peer review, offer diverse perspectives and framework on how stigma operates in various contexts and propose interventions that can address these challenges.

Becker et al. present a conceptual framework aimed at reducing stigma associated with Medications for Opioid Use Disorder (MOUD) by implementing structural changes in emerging acute substance use service models. The authors argue that stigma toward MOUD is a significant barrier to treatment engagement and propose that integrating MOUD initiation into acute care settings, such as emergency departments, can help normalise its use and reduce associated stigma. The study identifies three key processes—community outreach with peer influence, clinical evaluation and induction of MOUD in acute settings, and transition to outpatient care—as critical to mitigating stigma. The authors emphasise the importance of structural changes that empower patients and support their pursuit of life goals. This article underscores the need for empirical research to test the proposed framework and assess its effectiveness in reducing stigma and improving patient outcomes.

Selbekk et al. explore the role of Recovery Colleges (RCs) in reducing stigma and supporting recovery for individuals with mental health and substance use challenges. Through qualitative interviews, the authors investigate the experiences of participants in RCs, highlighting how these non-clinical learning environments enable individuals to shift from stigmatised identities to empowered roles as students and trainers. The study finds that participation in RCs facilitates significant personal transitions, such as moving from institutionalised identities to seeing oneself as a whole person and from being a recipient of care to an active agent in one’s recovery. The authors argue that RCs provide an invaluable complement to traditional services by fostering autonomy, social connection, and resilience. This article contributes to the literature on stigma reduction by demonstrating the transformative potential of alternative recovery settings.

Patel et al. investigate the persistence of stigma among individuals who have ceased substance use compared to those who currently use substances. Using a survey conducted in Michigan, the authors examine five dimensions of stigma: enacted stigma, anticipated stigma, internalised stigma, social withdrawal, and treatment stigma. The study finds that while internalised stigma is significantly lower among those who have ceased substance use, other forms of stigma persist regardless of substance use status. These findings suggest that stigma is not solely tied to substance use behaviour but is deeply embedded in societal perceptions and attitudes. The authors argue that public health efforts to reduce stigma must address the needs of both current and former substance users to be effective. This article provides valuable insights into the complexity of stigma and the challenges of achieving lasting stigma reduction.

Pouille et al. address the intersection of stigma, structural inequity, and cultural competence in the treatment of substance use problems among individuals with an Islamic migration background. Through a co-creative case study, the authors blend academic literature with lived experiences to explore how the continuum of care for substance use can be tailored to meet the needs of this population. The study highlights the barriers to care faced by individuals with an Islamic background, including cultural insensitivity and structural inequities, and proposes culturally competent interventions that can bridge the gap between what is needed and what is available. The authors caution against the dangers of *culturalisation*, urging for a nuanced approach that avoids essentialising or othering individuals based on their cultural background. This article contributes to the discourse on stigma by emphasising the importance of culturally responsive care in reducing stigma and improving treatment outcomes.

Bach et al. offer a comparative analysis of smaller open alcohol and drug scenes in Denmark and Norway, exploring their characteristics, functions, and the role they play in the lives of marginalised individuals. Through fieldwork, including participant observation and interviews, the authors examine how these spaces, often centred around shed-like structures, provide informal care, community, and a sense of decency among users. The study finds that these smaller scenes, though unregulated, are spaces of ambivalence where marginalised citizens negotiate their existence and find support. The authors argue that these scenes can reduce harm for marginalised individuals by offering a place of refuge and community. However, they also emphasise the importance of dialogue between users and the wider community to reduce stigma and foster understanding. This article expands the discourse on stigma by highlighting the nuanced ways in which marginalised groups create and navigate their social spaces.

The articles in this RT collectively advance our understanding of the multifaceted nature of stigma and its impact on individuals with SUDs. Theoretically, these studies underscore the importance of addressing stigma at multiple levels, from individual perceptions to structural and cultural frameworks (6). Practically, they offer evidence-based strategies for reducing stigma, including integrating stigma reduction into service models, creating alternative recovery settings, and fostering culturally competent care. These findings also highlight the need for ongoing research to explore the nuances of stigma across different populations and contexts, as well as the effectiveness of various interventions. Policy implications include the need for structural reforms that integrate stigma reduction into healthcare policies and practices, ensuring that individuals with SUDs receive equitable and compassionate care. Additionally, the importance of culturally responsive care cannot be overstated, as addressing the specific needs of diverse populations is crucial for reducing stigma and improving treatment outcomes.

This RT advances our understanding of stigma and its impacts, contributes to the academic discourse, and has the potential to inform practical applications for research, advocacy, and intervention to reduce stigma and support the recovery and well-being of individuals with SUDs. The Editors would like to extend their sincere appreciation to all the authors, reviewers, and editorial board members who contributed to this RT. Their collective efforts have created a significant resource for researchers, practitioners, and policymakers dedicated to breaking the cycle of stigma and improving the lives of individuals with SUDs.
